# Imaging in Non-neurologic Oncologic Treatment Planning of the Head and Neck

**DOI:** 10.3389/fvets.2019.00090

**Published:** 2019-03-28

**Authors:** Katherine S. Hansen, Michael S. Kent

**Affiliations:** Department of Surgical and Radiological Sciences, UC Davis School of Veterinary Medicine, Davis, CA, United States

**Keywords:** CT, MRI, radiation, chemotherapy, surgery, veterinary, oncologic, cancer

## Abstract

Imaging is critical for the diagnosis and staging of veterinary oncology patients. Although cytology or biopsy is generally required for diagnosis, imaging characteristics inform the likelihood of a cancer diagnosis, can result in a prioritized list of differentials that guide further staging tests, and assist in the planning of surgery, radiation, and chemotherapy options. Advanced imaging, such as CT and MRI, can better define the extent of disease for surgical and radiation planning for head and neck cancer cases in particular. Additionally, new imaging technologies are continually being translated into veterinary fields, and they may provide more options for cancer patients as they become more widely available.

## Patient Staging

Imaging can be used to assess the extent of local disease and response to treatment, identify lymph nodes that may have metastatic disease, screen distant organs for possible metastasis, and allow image-guided sampling of suspected neoplastic lesions. Choosing the best imaging technique for a patient depends on several factors including where and what is being imaged, what information is needed, and also sometimes cost of the imaging procedure to decide on a best course of action for an individual patient. It should be noted, however, that while imaging can be suggestive of cancer, no imaging technique is able to diagnose cancer *per se* on its own, and that a histopathologic or cytologic diagnosis is required for confirmation.

Cancer staging refers to determining the local extent of the tumor and whether or not it has spread. While multiple staging schemes for individual tumor types in different locations have been developed in veterinary medicine, they have not always been shown to provide prognostic value, and in some cases multiple staging schemes have been proposed for a particular tumor type. The concept of staging however is essential to developing a treatment plan for a particular patient, and imaging is central to staging a patient. Generally, tumor staging follows the World Health Organization's recommendations, which are based on the TNM system (1). T refers to the local tumor and relates to the size, and in some cases the invasiveness and/or the histologic grade, of the tumor. N relates to regional and distant nodal metastasis, and M refers to other, non-nodal metastasis of the tumor. In order to develop a rational treatment plan for a veterinary oncology patient, it is essential to gather as much information as possible about the tumor, its biological behavior and the patient. A biopsy when available will usually provide a histologic diagnosis and grade which can in turn guide decision making in how to approach the individual patient.

Staging of particular tumor types by location is discussed in the disease specific sections of this article below; there are however are some general considerations. There is a balance to be reached between thoroughly evaluating a patient and performing imaging studies that are likely to be of low yield, or even perhaps finding lesions that are not likely related and lead to unnecessary invasive procedures.

While most clinicians would agree that a general health screening, particularly for animals that will be anesthetized for imaging or other procedures, including a complete blood count, chemistry panel, and ideally urinalysis for an older animal diagnosed with cancer is appropriate, what is included in this screening beyond basic bloodwork is more controversial. In one study of dogs presenting to an oncology service, approximately 3% of the 1,722 dogs during the study period were diagnosed with multiple distinct malignancies (2). Cases diagnosed with thyroid carcinoma, malignant melanoma and mast cell tumor that also had at least one other type of cancer diagnosed were found to be overrepresented in the study group. All three of these tumor histologies can affect the head and neck. Interestingly the study authors also found that 1/3 of the dogs presenting with thyroid tumors were diagnosed with at least one additional tumor. A separate study looking at dogs diagnosed with soft tissue sarcomas, primary brain tumors, and nasal tumors that had thoracic radiographs and abdominal ultrasound done as part of their staging also found a 3% rate of multiple distinct malignancies (3). A third study that retrospectively evaluated the diagnosis of additional synchronous tumors discovered during computed tomography (CT) scans performed for radiation therapy planning also found an occurrence of approximately 3% in 736 studies (4).

Regardless of tumor type or grade of a tumor, some clinicians recommend an abdominal ultrasound of all patients prior to proceeding with advanced imaging studies or treatment. This is to evaluate the patient for concurrent disease and general health. In the Marchello study mentioned above, using abdominal ultrasound to screen dogs diagnosed with soft tissue sarcomas, primary brain tumors, and nasal tumors, all of which are reported to have a low metastatic rate on diagnosis, they found abnormal abdominal ultrasound characteristics in 78, 87, and 80% of the cases, respectively (3). In the majority of these cases the findings were not considered to be major, and only four of these findings in 101 cases altered the treatment plan.

More work needs to be done to evaluate the best practice with regards to a general health screening and what that includes. For now, offering an owner a more comprehensive work-up than might be required for a particular tumor type is reasonable as long as it is balanced with a discussion of cost vs. likely benefit. These costs include not only financial considerations but also the risk to the patients when the imaging procedure requires anesthesia.

**Figure 1:**
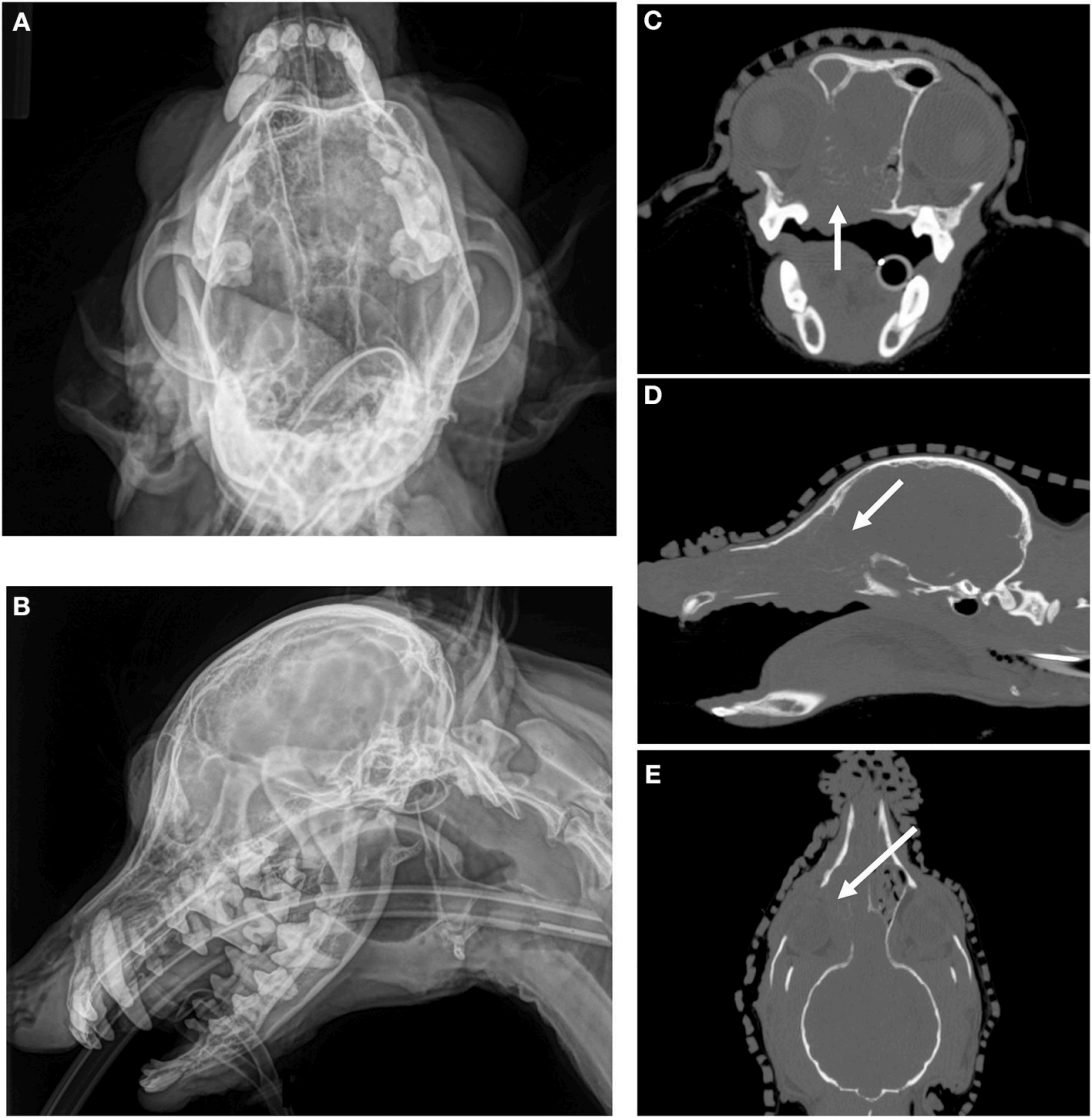
CT imaging provides superior assessment of bone destruction for a canine nasal carcinoma. **(A)** (Upper Left) and **(B)** (Lower Left) Oblique and lateral images reveal a mass in the right nasal cavity and sinus, but the degree of boney involvement is not well defined. **(C)** (Upper Right), Transverse, **(D)** (Middle Right) Sagittal, and **(E)** (Lower Right) Dorsal images of the same dog with non-contrast CT imaging reveals extensive, right-sided bone destruction of the hard palate (white arrow, **C**), cribriform plate (white arrow, **D**), turbinates and orbit (white arrow, **E**) that is not clearly evident on the original radiographs.

**Figure 2:**
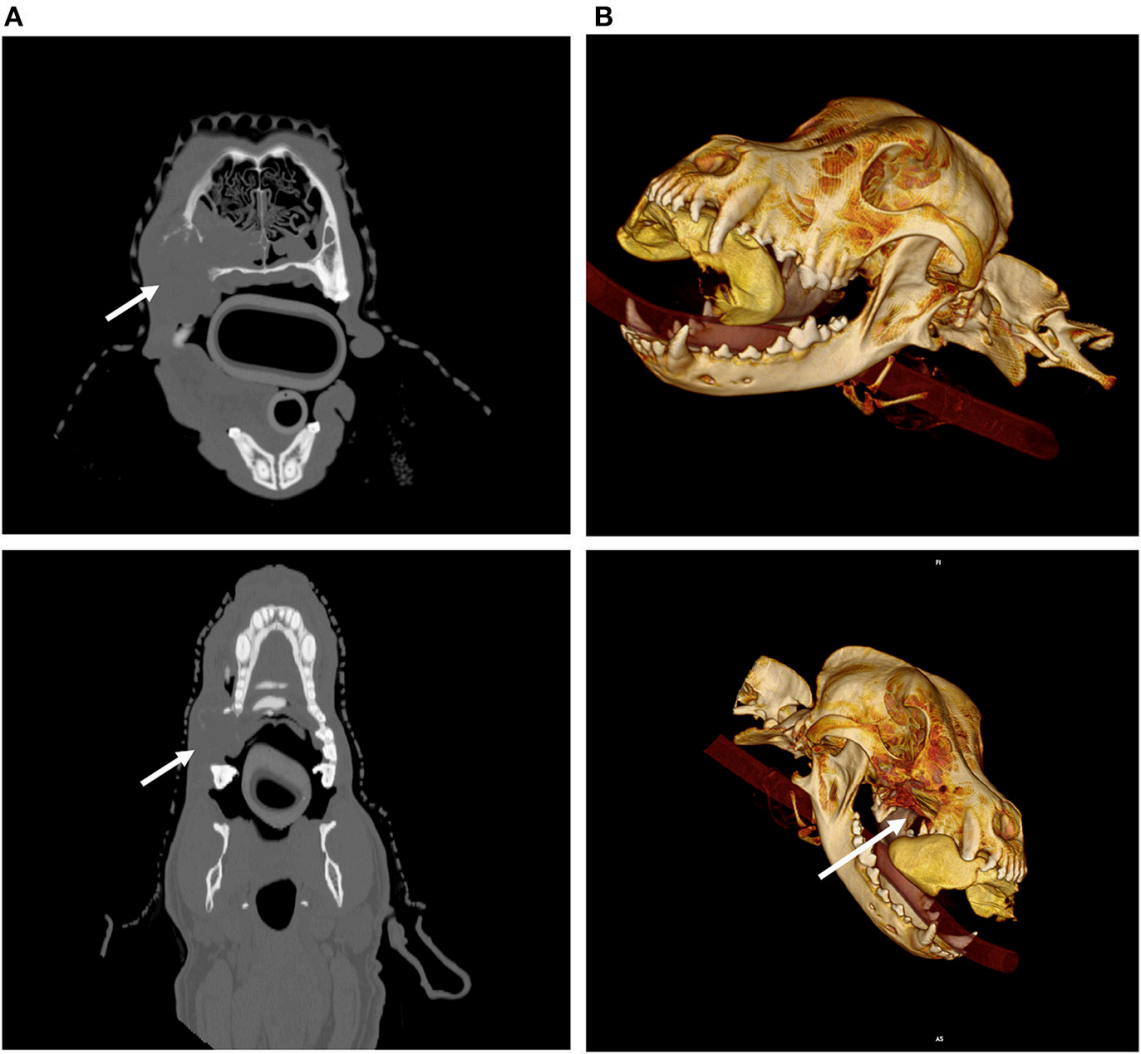
Imaging and radiation planning for adaptive radiotherapy. **(A)** (Upper left) Original radiation planning CT image with PTV (red line and yellow-shaded region) contoured for a nasal carcinoma. **(B)** (Upper right) Mid-way through radiation treatment the tumor decreased in size. The shrinkage caused the right eye (blue shaded region) to fall within the original PTV (red line and yellow-shaded region). The green shaded area shows the region of the eye now overlapping with the PTV, and is also noted with a solid arrow.

Imaging, particularly with CT and magnetic resonance imaging (MRI), can also be used when evaluating the local extent of disease to help determine if a tumor is resectable and to help plan the surgical dose needed to attempt a complete resection (5). CT in general is considered superior than standard radiography in identifying tumor, underlying boney change as well as invasion into adjacent structures (6) as noted in Figure 1 with a canine nasal carcinoma. MRI can also help delineate the extent of tumor and whether a complete resection is possible. The role of positron emission tomography (PET/CT) imaging in defining the true extent of tumors is still very early in development in veterinary medicine, although it holds promise of helping to more accurately define tumor extent which in turn can help surgeons plan their treatments (7). Additionally, 3-dimensional (3D) reconstructions of CT scan images can help surgeons assess whether surgery is a possibility for head and neck tumors (Figure 2). 3D reconstructions also help surgeons map locations for osteotomy and can provide opportunities for 3D-printed prostheses (8, 9) For example, a recent study combined CT imaging, 3D reconstruction, and a regenerative surgical bone matrix to improve post-operative outcomes in five dogs receiving rostral manibulectomy (10).

**Figure 3:**
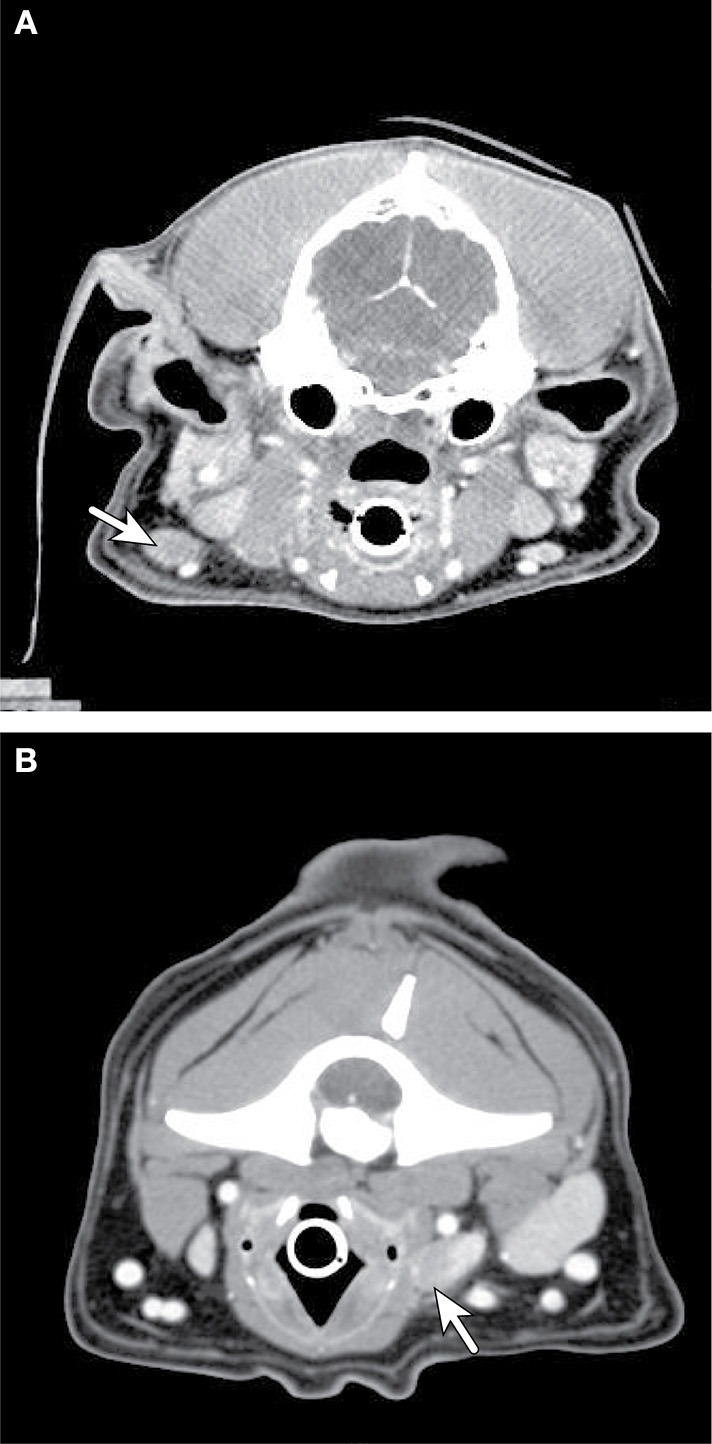
Axial CT imaging of metastatic neck lymph nodes associated with canine oral tumors. **(A)** (Top) Melanoma metastasis was detected in the enlarged, contrast enhancing, left mandibular lymph node (solid arrow) on CT imaging. **(B)** (Bottom) Metastatic melanoma cells from an oral mass were found in the enlarged, heterogeneously contrast enhancing, right retropharyngeal lymph node (solid arrow) on CT imaging.

When evaluating head and neck tumors for locoregional metastasis, the major regional lymph nodes to identify include the mandibular, medial retropharyngeal, cervical, and superficial cervical lymph nodes (11) (Figure 3). Uncommonly the facial nodes, parotid lymph nodes and the lateral retropharyngeal lymph nodes may be affected. It is important to evaluate all the lymph nodes and not just the mandibular ones as over 50% of the cases with regional nodal metastatic disease in head and neck cancer may not involve the mandibular lymph nodes (11). CT is commonly used to identify lymph nodes in head and neck cancer cases. Its value in determining if there is metastasis is limited, however. CT and ultrasound can also be used to guide aspirates and sampling of lymph nodes to assess them for metastatic disease. One study looking at the diagnostic accuracy of CT for determining metastatic mandibular and medial retropharyngeal lymph nodes in dogs with either nasal or oral cancers found sensitivity was 12.5 and 10.5%, and the specificity was 91.1 and 96.7%, for mandibular and medial retropharyngeal lymph nodes, respectively (12). This same study found that no CT parameter was predictive of metastasis. In another study, 23% of enlarged lymph nodes were identified as metastatic and 6% of normal appearing lymph nodes on CT were aspirated and contained metastatic disease (4). These studies highlight the importance of sampling lymph nodes even if they appear normal on imaging.

Imaging can also be used to help identify the sentinel lymph node (first draining lymph node) or nodes from a tumor bed. This is ideal as it focuses the clinician to evaluate only these sentinel nodes rather than all of the regional nodes (13). There have been multiple studies using imaging techniques to identify sentinel lymph nodes from primary tumors in dogs. These techniques include CT lymphography, lymphoscintigraphy, and contrast ultrasound (14–16). Again, these techniques are used to simply identify the draining lymph nodes, but these lymph nodes still must be sampled either by aspirate or biopsy to determine if there is metastatic disease present. Novel imaging techniques such as PET/CT are also on the horizon and have been used to identify locoregional lymph node involvement (17).

Screening the lungs for possible metastasis is common practice for most malignant head and neck tumors. Again, some clinicians recommend this imaging procedure for all patients prior to invasive procedures and as part of a general health screen, particularly for geriatric animals regardless of the tumor diagnosis. Three view thoracic radiographs taken at peak inspiration are the generally accepted minimal standard for assessing the lungs for both a general health screening and for screening for metastatic disease (18–20). The likelihood of discovering metastatic disease is dependent on the biological behavior of the tumor histology and grade. Although even in tumors that are thought not to be highly metastatic, its presence would likely change the treatment plan. Other non-neoplastic disease can also be identified, which can justify carrying out this imaging procedure (3). More recently CT has been used to assess veterinary subjects for the presence of pulmonary metastasis and has been found to be more sensitive than 3-view thoracic radiographs in detecting pulmonary metastasis (21–23). One of these studies suggested that pulmonary nodules of 1 mm could be detected on CT while nodules had to be at least 7–9 mm for detection using conventional thoracic radiographs (23). However, because few data exist on the outcomes expected when lung involvement is detectable on CT imaging but not radiographs, it is not clear to what degree a lead-time or length-time bias may play a role as more data are collected (24).

Whole body CT scans have fallen out of favor in human medicine for cancer screening as it has not been shown to be an effective screening method. However, whole body CT has been effectively used for staging patients already diagnosed with cancer. There are limited studies in veterinary medicine on using whole body CT to screen dogs and cats with a cancer diagnosis. Studies describing this procedure to date have been limited and included findings of muscle metastasis in whole body CT scans in dogs and cats and for staging of osteosarcoma (25). Fused PET/CT has been used more commonly in human medicine for cancer staging than whole body CT alone and is starting to be explored in veterinary medicine.

## General Approach to the Oncologic Patient

### Treatment Approaches: Surgery, Chemotherapy, and Radiation

#### Multi-Modality Therapy

Malignant tumors require both local control and control of possible regional or distant metastasis to achieve the best patient outcome. Surgery remains a mainstay of treatment for local control for many tumors of the head and neck. Tumors that cannot be completely resected may also be treated with radiation in some cases. One exception to surgery as a primary option is in the treatment of nasal tumors. Most nasal tumors, regardless of cell of origin, are treated with radiation therapy (26).

Head and neck tumor types carrying significant metastatic potential may also be treated with chemotherapy in addition to surgery or radiation. For smaller canine oral melanomas, clinicians may recommend primarily surgery, or surgery followed by radiation, and also offer the option of carboplatin and/or the melanoma vaccine after local control to help reduce the risk of metastasis (27–29). Thyroid tumors are another example where a combination of surgery, radiation, and chemotherapy may be recommended depending on the expected risk for metastasis and the surgical outcome (30, 31). There are more data regarding outcomes for some tumors than others, and it can be challenging to make strong recommendations for a particular combination of treatments based on the number of veterinary cases in the literature (26, 32). The concept of chemoradiation has also been attempted in veterinary medicine, wherein chemotherapy and radiotherapy are delivered together with the intent to radiosensitize cells with a chemotherapy drug and improve tumor control. However, radiosensitization can also increase normal tissue toxicity and bone marrow suppression, resulting in undesirable breaks in treatment (33).

#### Imaging for Radiation Planning

Modern radiation oncology is heavily reliant on imaging for treatment planning. Radiation oncologists will identify and map out target volumes and critical normal structures with the idea of maximizing dose to the tumor and minimizing dose to the surrounding normal structures. The three main tumor target volumes that are used for prescribing a dose are the gross tumor volume (GTV), the clinical tumor volume (CTV) and the planning target volume (PTV). The GTV is the defined as the visible tumor on visual inspection or imaging. The CTV comprises the GTV with the addition of a margin, dependent on the tumor type, to account for subclinical and microscopic spread of the tumor into surrounding tissues, and the PTV is an additional margin that is added to the CTV to account for errors in positioning, patient motion during treatment, and machine and imaging alignment on the therapy unit. With the advent of image guidance with electronic portal imaging, and linear accelerators with built-in Cone Beam Computed Tomography (CBCT) or other 3-dimensional imaging, the PTV can be reduced, but not generally eliminated, allowing for improved conformality of dose. Determination of an individual patient's GTV and CTV, while fairly straight forward to define in concept, can in actuality be difficult to delineate and contour on imaging sets (34). Functional imaging such as PET/CT scanning may allow better delineation of the GTV and possibly CTV, although its use in veterinary medicine remains largely unexplored (7).

**Figure 4:**
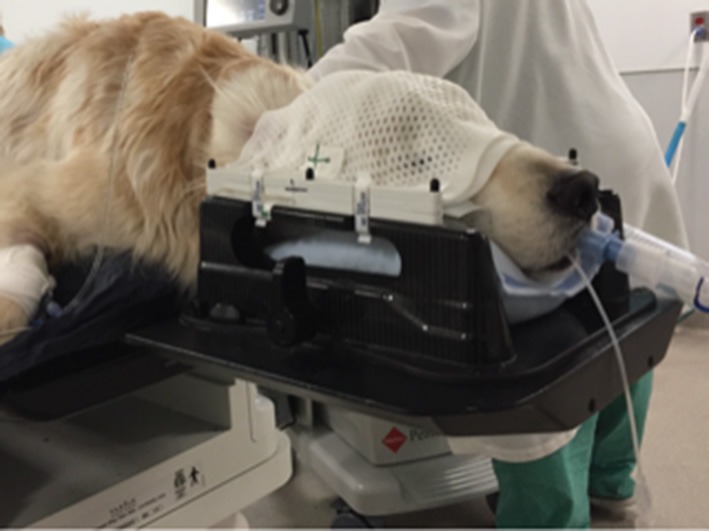
Canine patient with head and neck positioning device. An anesthetized canine patient is placed in a positioning frame with pillow and thermoplastic mask used for head and neck radiation patients.

CT scans are specially adapted for radiation planning. Although many human patient facilities have a dedicated CT scanner for so-called radiation planning “simulation CT” scans (35), veterinary hospitals often have removable, flat couch-tops that can be utilized on the hospital CT scanner that is otherwise used for diagnostic CT imaging cases (36, 37). Duplicating the setup used for the CT simulation of radiation planning is critical for 3D radiation treatments. Thermoplastic masks, vacuum-locked moldable bags, dental molds for bite blocks, and nonmigrating fiducially imbedded in tumors aid in replicating the positioning of the patient and the PTV for subsequent treatments (38–46) (Figure 4).

The field of view of a CT scan for radiation planning is generally much larger than that routinely used for diagnostic imaging. As such, there are limitations as to how large of a patient can be treated based on the bore size of the CT scanner (47, 48). Additionally, the larger field of view results in lower resolution of the image, especially in small patients with large fields of view that may include the shoulders for small head and neck lesions (49). The slice thickness and subsequent volume averaging that occurs across the individual CT image can lead to over- or underestimation of a gross tumor margin with loss of spatial resolution as well. This may require that additional scans with small fields of view and relatively thin slice thickness are performed and then subsequently fused to the calculation CT to have better tumor delineation.

Once images are acquired, they are imported into treatment planning software. In general, the CT images from which the radiation therapy treatment planner calculates should come from a non-contrast CT scan. As iodinated contrast agents are present at the time of imaging, but not when a patient is receiving radiation treatment, there is some concern that the additional attenuation seen on the contrast CT images could lead to dose calculation inaccuracies. This would be of particular concern near organs with a substantial amount of contrast uptake. In areas without a lot of contrast uptake by the local tissues, the dose calculated for a post-contrast CT image set is unlikely to vary by more than 1% compared to a calculation performed for a pre-contrast CT image set (50).

## Selected Tumor Types and the Role of Imaging in Diagnosis and Treatment

### Oral Tumors

The most common non-odontogenic oral tumors seen in veterinary patients include oral melanoma, squamous cell carcinoma, and sarcomas. The most common odontogenic oral tumors seen in veterinary patients include those formerly called epulides, now referred to as acanthomatous ameloblastomas and peripheral odontogenic fibromas.

**Figure 5:**
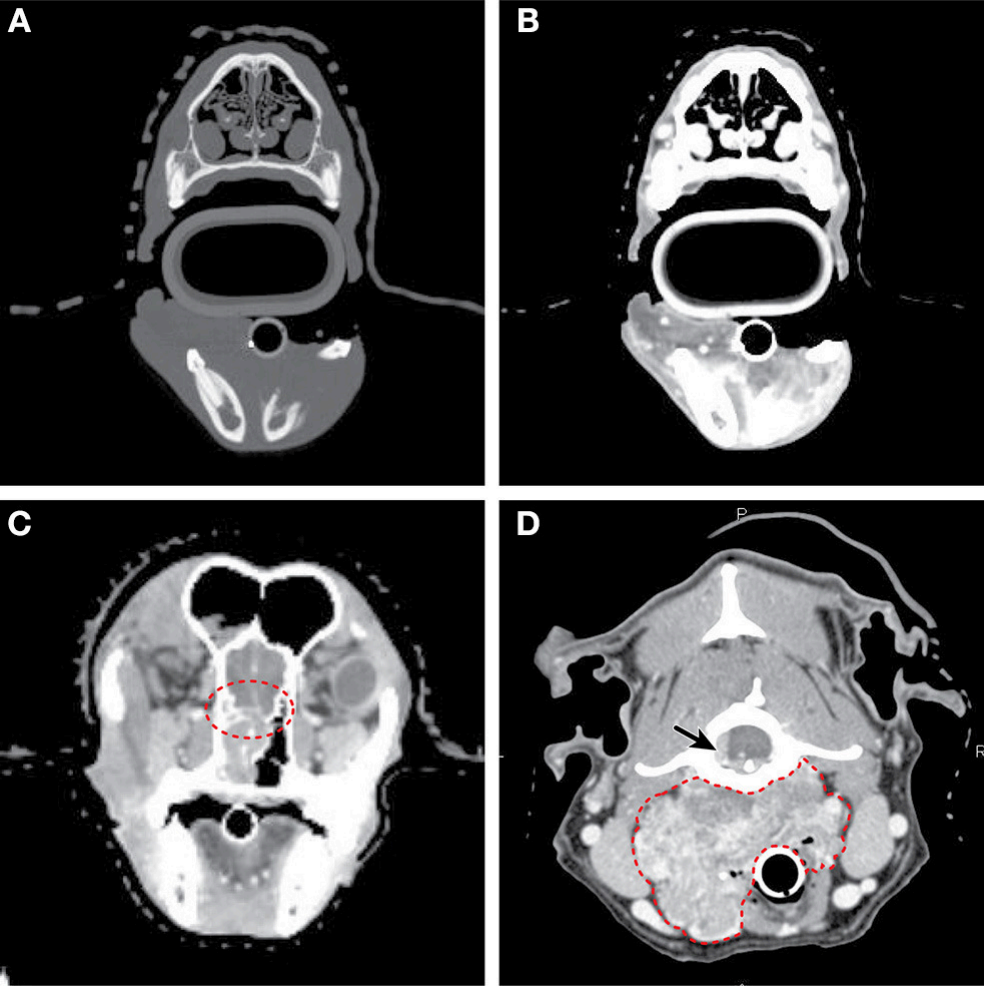
Axial CT imaging of primary head and neck tumors. **(A)** (Upper left) Non-contrast CT imaging with bone window/leveling reveals extensive bony lysis in one mandible from an oral melanoma. **(B)** (Upper right) Contrast CT imaging of the same case reveals a heterogeneously contrast enhancing mass that extends medially into the soft tissues near the affected mandible. **(C)** (Lower left) Contrast CT imaging reveals cribriform lysis (red circle) and invasion into the cranial vault of a contrast enhancing nasal carcinoma. **(D)** (Lower right) A large, contrast enhancing, carotid body tumor infiltrates into the surrounding soft tissues (dashed red line) and extends to the spinal cord (solid arrow).

CT can provide anatomic information and 3D-spatial information for oral tumors that is superior to radiography. CT is often necessary to define the extent of more caudally located tumors for surgery and radiation planning, and CT can better define regional lymph nodes in terms of size when compared to ultrasound (51). Both CT and MRI may be important for assessing the extent of disease and potential bone involvement, improving the chance of a complete excision and better outcome (6, 52) (Figures 5A,B). Recently, a study on oral tumors in dogs showed that while MRI and CT showed similar findings for bony changes, CT was better for visualizing calcification and cortical bone destruction, while MRI was better to evaluate tumor size and invasion of adjacent soft tissue structures (53). When comparing conventional radiography and CT, the cross-sectional imaging of CT avoids superimposition of tissues, and in one study of 21 dogs, bony changes were detected more commonly on CT when compared to radiographs (95.2 vs. 80.9%). Invasion into neighboring structures was also much more readily detected with CT (90.4 vs. 30%) in the same study. Additionally, it is reported that bone lysis is not evident on plane films until 40% or more of the cortex is destroyed (54).Therefore, CT appears to be critical for surgical and/or radiation therapy planning for oral cancers (6).

Additionally, regional lymph nodes and their contrast uptake can be assessed with advanced imaging; however, in a recent study the sensitivity of CT for detecting mandibular and medial retropharyngeal lymph nodes metastasis was low (10.5–12.5%), specificity was high (91.1–96.7%), and accuracy was modest (67.5–76.3), suggesting that relying on CT imaging alone for lymph node diagnosis is insufficient (12). Moreover, the metastatic pattern of oral tumors can be variable: for example, 40% of dogs with normal-size lymph nodes had melanoma metastasis (55), and only 55% of oral cancer patients with metastasis to a regional lymph node having metastasis to the mandibular lymph node (11).

#### Canine

Oral cavity tumors are the 4th most common cancer in dogs (56). Canine oral tumors are generally locally invasive and the chance of metastasis depends on the histology, with some canine squamous cell carcinomas and most oral canine malignant melanomas spreading to locoregional lymph nodes and the lungs (56).

##### Melanoma

Melanoma is most common oral cancer in dogs, and is seen on the gingiva, lips, tongue, and hard palate (56). Oral melanoma behaves more aggressively than its haired skin counterpart, and size and stage inform survival in these patients (57, 58). Because melanoma is highly metastatic and also locally invasive, including into bone, CT imaging can be highly valuable for surgical and/or radiation planning.

##### Squamous cell carcinoma

Squamous cell carcinoma (SCC) is the second most common oral tumor in the dog (59). SCC can also invade into bone, and as such CT imaging is helpful in defining the extent of disease. Non-tonsillar SCC in dogs appears to have a modest metastatic rate of approximately 20% (60).

##### Sarcoma

Fibrosarcomas are the third most common oral malignancy in the dog, and often occur in large breed dogs. As a group, they are locally invasive but with a moderate lung metastatic rate of <30% (60, 61). A specific subtype of fibrosarcomas referred to as histologically low-grade but biologically high grade, have a remarkably benign appearance on histology similar to fibromas, but can be rapidly growing and readily invade into bone (62). Advanced imaging with CT can be critical to plan for appropriate surgical margins, provide realistic expectations for surgical cure, and prepare for radiation planning of these oral fibrosarcomas (61).

##### Epulides

Epulides are considered benign masses, and they include acanthomatous ameloblastomas (previously known as acanthomatous epulis) and peripheral odontogenic fibromas (previously known as ossifying and fibromatous epulides) (63). Acanthomatous ameloblastoma has the unique feature among epulides of frequently invading into underlying bone. This bony invasion can be better defined by CT imaging to prepare for larger resections and/or radiation fields required for this tumor type compare to other epulides (64, 65).

#### Feline

Oral cavity tumors comprise approximately 3% of all cancers in the cat, with, squamous cell carcinoma being the most common oral malignancy. SCC in cats may invade extensively into bone in cats such that CT imaging is of great benefit (66). Metastatic frequency is not completely defined because local control is uncommon, resulting in euthanasia due to the primary tumor after a short period of time in many cases (67). Fibrosarcomas are the second most common tumor in the cat (56), but comprise <20% of feline oral tumors (68). Metastasis is relatively uncommon, and treatment involves primarily surgery or radiation. Epulides are uncommon in cats, with a clinical presentation of multiple epulides being noted, particularly in young cats (69).

### Intranasal

CT provides precise anatomic information that is superior to radiography for both oral and nasal tumors when compared to radiographs (70). A staging system has been developed based on CT imaging as well, where the integrity of the cribriform plate may be associated with outcome (71) (Figure 5C), but several other staging systems have also been proposed.

On MRI and CT, bony destruction, sphenoid sinus destruction, retrobulbar space involvement, nasopharyngeal involvement, lateral maxillary hyperostosis, and patchy increased density within abnormal soft tissues are associated with, but not definitive for, nasal cancer (72, 73). Importantly, a mass in the nasal cavity is not specific for nasal neoplasia (72). While CT is most commonly used for imaging the nasal cavity when staging nasal tumors, there have been two studies comparing their utility to MRI. In one study, comparison of MRI and CT revealed that MRI is only more helpful in defining the portions of tumors that extend into the cranial vault (74). In another more recent pilot study evaluating the utility of each imaging modality for the staging of nasal tumors, there was a high level of agreement when assessing bone involvement. However, MRI revealed a higher tumor volume in five out of six cases and was also better at detecting meningeal enhancement than CT (75). Regarding radiographs of the nasal cavity, similar to advanced imaging, they must also be obtained with the patient under general anesthesia to allow for precise positioning needed to obtain a diagnostic study (76). Radiographs are only similarly sensitive to CT for detecting changes associated with nasal tumors in cases that present with clinical signs (77). However, they are of limited use in determining the extent of disease.

#### Canine

Nasal tumors are more common in dogs than in cats, and carcinomas (comprising approximately 2/3 of cases) are more common than sarcomas in dogs, with nasal lymphoma being rare in canines (78). Nasal tumors are locally invasive, and extension into the brain may occur in advanced cases. Although patients are usually euthanized due to signs related to the primary mass, metastasis has been noted in up to 50% of cases at the time of death, most commonly to the draining lymph nodes and to the lungs (56).

CT is useful for staging of cases although the staging systems remain controversial. The Adams staging system with stage 4 (cribriform plate involvement) correlates with outcome (71). However, recent studies using stereotactic radiation for cases with cribriform involvement did not associate this CT finding with outcome (79). CT can also be used to assess for response to treatment and recurrence, as evidenced by 46% of cases having marked tumor regression in one study (80) and approximately 86% in another study (81).

#### Feline

Lymphoma is the most common nasal tumor of the cat (56). Carcinomas, including SCC are the next most common category, with sarcomas and other tumors less commonly reported (82, 83). CT may reveal paranasal bone osteolysis, extension into the orbit, a space-occupying mass, or turbinate destruction that may be more common with neoplasia (84), but ultimately severe rhinitis can result in similar signs. Another study suggested that feline intranasal neoplasia was more likely in cases with unilateral lysis of the turbinates, lysis of a variety of bones including the dorsal and lateral maxilla, vomer bone, and ventral maxilla, and soft tissue or fluid in the sphenoid recess, frontal sinus, or retrobulbar space (85). Evaluation of the cribriform plate for extension of tumor into brain may also be prognostic (86). Finally, several institutions are exploring both bland embolization and chemoembolization procedures using CT guidance for nasal tumors in dogs and cats, with one successful treatment reported in a cat with adenocarcinoma (87).

### Ear Canal

#### Canine

Ceruminous gland tumors (both benign and malignant) are the most common type arising from the ear canal, with SCC, undifferentiated carcinomas, and several other tumor types being reported in the literature as well. There are conflicting data as to whether benign vs. malignant ceruminous gland tumors are more common in the dog, which cannot be differentiated on advanced imaging (88, 89). Because a significant subset of canine ear canal tumors are ceruminous gland adenocarcinomas that can metastasize eventually and are also locally invasive (for example 13/27 dogs in one study had bulla lysis on CT), CT is recommended for surgical planning and assessment for locoregional disease (88). Additionally, bilateral cancer has been reported in the dog (90). CT scans can also be used for radiation planning (91).

#### Feline

Inflammatory polyps are seen commonly in cats, with benign ceruminous gland adenomas also reported in multiple cases (88). Ceruminous gland adenocarcinomas are the most common malignant ear canal tumor in the cat, and are more common than their benign counterpart (89). Additionally, cats can develop SCC that can be highly invasive into bone and surrounding tissue (88). Because a significant subset of feline ear canal tumors is either ceruminous gland adenocarcinoma or SCC that can metastasize and are locally invasive, CT is recommended for surgical planning and assessment for locoregional disease.

### Skull

#### Canine

##### Osteosarcoma

Axial osteosarcomas are less common than appendicular locations; they comprise only about one-quarter of all cases and have a lower metastatic rate than appendicular tumors (92). Unlike appendicular tumors where amputation is often an option, local tumor control remains a major hurdle with axial tumors, even with modern surgical options. Complete surgical margins are important for overall prognosis, with one study not reaching a median disease free interval (DFI) even with 1,503 days of follow up after complete excisions of skull osteosarcomas (93). In another study where patients were treated with a combination of radiation, surgery, and/or chemotherapy, the cause of death was tumor recurrence in more than half of cases (94).

Based on information on appendicular osteosarcoma tumor imaging, MRI images overestimated the intramedullary extent of osteosarcomas compared to gross and histologic disease by only 3%, with T1-W non-contrast images being sufficient in most cases. In comparison, lateromedial and craniocaudal radiographs overestimated tumor length by 17 and 4 %, respectively. Scintigraphy and CT overestimated tumor margins by 14 and 27%, respectively (95). In another study, radiography was more accurate than nuclear scintigraphy at measuring the extent of distal radius osteosarcomas, as nuclear scintigraphy overestimates tumor length (96). Importantly, CT appears to provide overall more accurate extent of disease, with good correlation between histopathology and imaging of intramedullary/endosteal abnormalities with a mean overestimation of 1.8% (*SD* = 15%). The length of abnormal contrast enhancement had a mean overestimation of 9.6% (97). Radiography may underestimate tumor extent more often than advanced imaging techniques (98). Together these data indicate that CT may be the best imaging modality to assess bone involvement for surgical planning.

##### Multilobular tumor of bone

This uncommon tumor type can arise from various locations on the skull, including both the mandible and maxilla, hard palate, orbit, nasal cavity and calvarium (99). These tumors have a characteristic, but not necessarily pathognmonic, CT imaging feature of a “popcorn” appearance defined by rounded, well-defined lesions with either fine or coarse granular, nonhomogeneous bone (100). MRI can also be beneficial for those tumors abutting CNS tissues, and the tumors have mixed signal intensities with significant areas of contrast enhancement on post-contrast T1-W images, and the extent of brain and soft tissue involvement is well-delineated for surgical planning (101). The recurrence rate for these tumors after surgery is relatively high and worse for high grade tumors (78% recurrence for Grade III), with moderate metastatic spread most commonly to the lung (99).

### Neck

#### Canine

##### Thyroid carcinoma

Thyroid tumors are relatively uncommon, with carcinomas being the most common type in the dog (102). In contrast, adenomatous hyperplasia is more common in the cat (103). In dogs, carcinomas are locally invasive and can spread via both lymphatic and hematogenous routes; therefore, metastatic disease is commonly seen in the regional lymph nodes, lungs, and occasionally other locations (30, 104).

Ectopic thyroid tissue is commonly reported; it can be found from the base of tongue to the base of the heart (30, 105). Scintigraphy results in concentration of sodium 99mTc pertechnetate in regions of functional thyroid tissue, which can be helpful to identify ectopic thyroid tissue and metastatic disease, and this imaging technique is variably used for I-131 dosing in cats and dogs with functional thyroid masses (56). Scintigraphy may reveal heterogeneous or multinodular regions in feline carcinomas, which are uncommon tumors, but ultimately these scintigraphic findings are not specific to malignant thyroid disease (106, 107).

Ultrasound is more commonly available and is routinely used to assess thyroid masses in dogs. Fine-needle aspirate, when attempted, may benefit from ultrasound guidance for diagnosis. Neck lymph nodes can also be assessed with ultrasound (76). Ultimately, CT and MRI have been shown to more reliably identify margins of thyroid tumors in dogs for pre-operative planning (108) and CT can be used to plan for radiation therapy.

##### Tonsillar and pharyngeal tumors

*Pharynx* In a study with 25 pathologically confirmed pharyngeal neoplasias, including sarcomas, carcinomas, melanomas, and one lymphoma, CT features could not be used to predict pharyngeal tumor type in dogs, but were helpful for assessing mass extension, lymph node involvement, and distant metastatic spread (109), which would allow for either surgical or radiotherapy treatment planning.

*Tonsillars* Squamous cell carcinoma is the most common tonsillar cancer and is highly metastatic, with melanoma and lymphoma being the next most common. CT features from 14 dogs with cytology or histology diagnosis, was able to differentiate neoplastic from non-neoplastic tonsillar diseases. Regional lymph node CT features were useful for diagnosis in some cases, because nodes >18 mm, heterogeneous contrast and loss of the hypoattenuating hilus in medial retropharyngeal lymph nodes were common in tonsillar neoplasia. However, several dogs had little or no enlargement of the tonsil despite associated metastatic lymph node involvement (110).

*Carotid body tumors* The carotid body is a specialized organ that acts as a chemoreceptor, and tumors of the carotid body are uniquely located at the bifurcation of the common carotid artery. Brachycephalic breeds may have predisposition for forming this type of tumor (111). This tumor type can be confused with thyroid carcinomas on physical exam and imaging, but CT and MRI may be beneficial in differentiating this neck neoplasm, as most appear as a large mass centered at the carotid bifurcation with variable margination (Figure 5D). For both CT and MRI, carotid body masses are strongly and heterogeneously contrast enhancing. Interestingly, the basilar portion of the skull can be affected with this tumor type as well (112), and although the metastatic rate is relatively low, the tumor can spread to multiple sites (113).

#### Feline

##### Thyroid tumors

Multinodular adenomatous hyperplasia is the most common cause of hyperthyroidism in cats, while carcinomas comprise approximately 1-3% of hyperthyroid cases (114). Carcinomas are more locally invasive and are reported to metastasize in the majority of cases to the lymph nodes and/or lung (115). Tc-pertechnetate scintigraphy can be used to evaluate the extent of functional thyroid tissue for diagnosis and for planning for surgery, I-131 treatment, or external beam radiation. It is also helpful for identifying ectopic thyroid tissue and metastatic thyroid carcinoma tissue (107). Ultrasound is also useful to guide percutaneous ethanol injection for treatment of solitary adenomas in cats (116).

##### Tonsillar tumors

Cases of feline tonsillar tumors, most commonly squamous cell carcinoma, are not fully characterized in the literature, although cases are reported alongside oral squamous cell carcinoma cases in several studies with variable outcomes (117–119). This disease has the potential to spread to lymph nodes in a similar manner to dogs (56). Similar to dogs, advanced imaging with CT may benefit for assessment in surgical planning and identification of abnormal lymph nodes; however, cytology is still critical for lymph node assessment (120).

## What's on the Horizon for Oncologic Imaging?

New imaging technologies are continually being translated into veterinary medicine. CT and MRI are now more widely available in private practice, and PET/CT is increasingly available in academic settings. Moreover, specialized MRI imaging and advanced image registration software are paving the way for adaptive radiation planning and for assessment of treatment response.

### PET-CT

PET images can be used to diagnose and stage cancer. PET utilized tracers such as 2-deoxy-2-18F-fluorodeoxyglucose (FDG) which can detect areas of increased sugar metabolism, or other markers associated with tumor activity. FDG-PET is very sensitive for regions of high metabolic activity, and consequently PET is more sensitive for metastatic lesion detection than other modalities (7). However, highly metabolically active regions are not specific to cancer, and these active regions can represent areas of inflammation. Therefore, cytologic or histopathologic diagnosis is still needed even when PET is utilized. PET images are created by use of radiopharmaceuticals that emit positrons. These positron particles travel a very short distance before encountering an electron, and this encounter results in two photons that travel in opposite directions from each other. The PET scanner can register these photons from which it can then create an image based on the origin of the photons (7).

PET/CT systems are now commonplace in human medicine, and in these systems the PET image and CT image are acquired at a single imaging session with the resultant images superimposed. Therefore, combined PET/CT units provide the sensitivity of PET to detect tumors with the anatomic detail of a CT scan. These systems also eliminate error associated with manual fusion of PET and CT images when done by a technologist or clinician. PET imaging combined with CT is a rapidly advancing field in veterinary medicine, although only a few institutions provide this imaging modality to date (7, 17, 121–125). PET/CT can be useful for finding the extent of disease with greater sensitivity than contrast CT in veterinary patients. For example, a recent report showed that cats with oral squamous cell carcinoma had more extensive soft tissue infiltration, with FDG-PET detecting areas of possible primary tumor that were not seen on contrast-enhanced CT scans (124). PET/CT is also used to target molecules relevant to tumor staging. For example, lesions consistent with disseminated hemangiosarcoma based on PET/CT were seen in 2/9 dogs, and these lesions had not been detected by radiography and ultrasonography (17). PET/CT may be useful to identify tumors, or regions of tumors, that may be more resistant to therapy. One group of investigators has shown changes in hypoxia PET markers in untreated tumors and in tumors undergoing radiation treatment (122, 123). However, PET has limitations, and highly metabolic areas that may be highlighted by FDG-PET are not specific for cancer because they may represent inflammation (7).

### DCE-MRI

Dynamic contrast-enhanced MRI (DCE-MRI) has recently been investigated in animals. It can be used for assessment of tumor perfusion in soft tissue sarcomas and brain tumors, and to follow tumor responses after hyperthermia and radiation therapy in animals (126).

### Image Registration for Radiation Treatment

#### What Is Image Registration?

Image registration involves alignment images from different data sets, for example images from CT, MRI and PET, or image sets from CT before and after treatment (127).

#### How Is Image Registration Used in Cancer Management?

Images acquired from CT and MRI are used throughout the radiation planning process, as previously described, from defining contours to verification of positioning for delivery of daily treatments (127, 128). In some cases, MRI, CT, or PET/CT images must be registered to each other so that they overlap for radiation planning, in order to better define targets or organs at risk in the radiation field. There are two types of registrations: rigid registrations where translations and rotations of structures that stay in spatial alignment to one another are matched, and deformable registrations where some degree of tissue motion can be matched between images. There are limitations to registration of images sets that have been reviewed elsewhere, and rigid registrations are generally limited to the head (127). Therefore, there may be some degree of organ motion even in the head and neck that must be accounted for in radiation planning simply based on the images being used (129). As defined above, target volumes contoured by radiation oncologists further take uncertainties about target extent and mobility into account in the volumes to be treated.

Registered images can also be used after treatments to confirm that the delivered dose is similar to the planned radiation dose. Recently, dose verification software that detects exit dose was assessed on a veterinary radiation system. This software relies on both proper registration of images used for treatment planning and proper registration of on-board images for treatment fractions (130).

**Figure 6:**
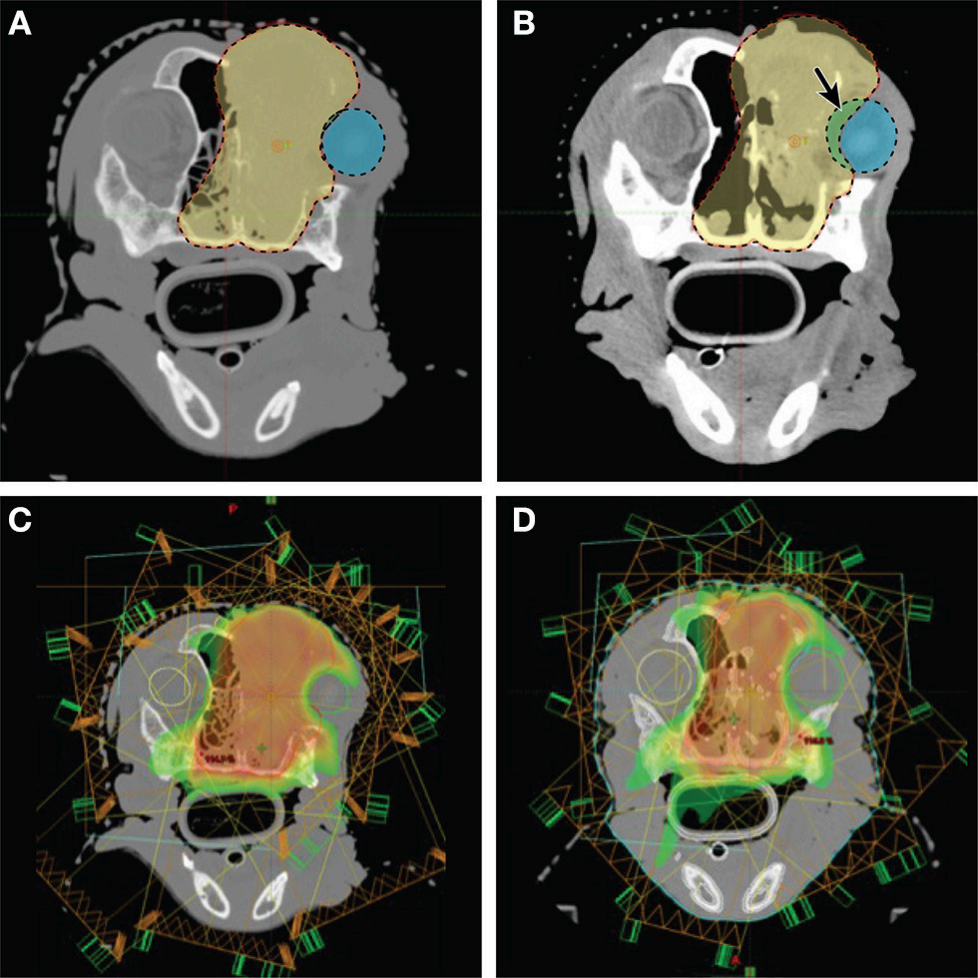
Imaging and radiation planning for adaptive radiotherapy. **(A)** (Upper left) Original radiation planning CT image with PTV (red line) contoured for a nasal carcinoma. **(B)** (Upper right) Mid-way through radiation treatment the tumor decreased in size. The shrinkage caused the right eye (blue shaded region) to fall within the original PTV (red shaded region). The purple shaded area shows the region of the eye now overlapping with the PTV, and is also noted with a solid arrow. **(C)** (Lower left) The original radiation planning heat map reveals regions of high dose (red/orange) and regions of moderate dose (yellow/green). The right eye (green circle) is originally planned to abut these dose regions. **(D)** (Lower right) A new “adaptive” radiation plan has been created to target the smaller, mid-treatment PTV. The right eye (green circle), is not only outside the PTV region, it is now farther away from the high dose (red/orange) and moderate dose (yellow/green) regions of the treatment field.

Image-guided radiotherapy (IGRT), can generally refer to using imaging to aid in radiation delivery. However, the term IGRT may specifically reference images used to aid in target contouring, especially when changes in the patient body (e.g., weight loss) or changes in the tumor (e.g., growth or regression) necessitate a modification to the radiation treatment plan. This type of IGRT is also known as adaptive radiation therapy (ART) (127). Adaptive radiation ultimately involves assessment of images during treatment, then registration of pre-treatment and during-treatment images to create a new “adapted” radiation plan as the contour of the patient or tumor changes during a course of radiation therapy (Figures 6A–D).

Because anatomy does not move spatially in a rigid manner (i.e., body parts and organs can move independent of one another based on patient position and normal tissue movements), there is limited use to rigid registrations for sites other than the head (127). Although not well described in veterinary medicine, deformable registrations can better help create efficient ART plans in the future (131).

### MRI-Based Radiation Planning

MRI is currently used, generally via rigid registration, to help with radiation contouring at many institutions (37, 132). MRI has superior soft-tissue contrast which is desirable for creating contours of normal tissues and tumors. There is interest in MRI systems that use 70 cm bores with a flat couch similar to those used for radiation treatment for treatment planning purposes. MR-simulators that no longer require a CT for planning are also highly desirable to simplify the radiation treatment planning process and limit CT radiation exposure. MR may also have a role for in-room positioning and target verification devices at the time of daily radiation treatments (rather than the currently popular CBCT), and this use of MRI would further limit the radiation dose associated with repeated CT imaging for patient treatment positioning (133).

## Author Contributions

All authors listed have made a substantial, direct and intellectual contribution to the work, and approved it for publication.

### Conflict of Interest Statement

The authors declare that the research was conducted in the absence of any commercial or financial relationships that could be construed as a potential conflict of interest.
